# Residential radon in Peru: challenges for health surveillance and lung cancer prevention

**DOI:** 10.17843/rpmesp.2026.433.16186

**Published:** 2026-09-14

**Authors:** Carol Ordoñez-Aquino, Diego Venegas Ojeda, Jhony A. De La Cruz-Vargas, Gustavo F. Gonzales

**Affiliations:** 1 Programa de Doctorado en Ciencias de la Salud, Facultad de Medicina, Universidad Nacional Mayor de San Marcos, Lima, Peru. Universidad Nacional Mayor de San Marcos Programa de Doctorado en Ciencias de la Salud Facultad de Medicina Universidad Nacional Mayor de San Marcos Peru; 2 Facultad de Ciencias e Ingeniería, Universidad Peruana Cayetano Heredia, Lima, Peru. Universidad Peruana Cayetano Heredia Facultad de Ciencias e Ingeniería Universidad Peruana Cayetano Heredia Lima Peru; 3 Facultad de Medicina, Universidad Nacional de Ucayali, Pucallpa, Peru. Universidad Nacional de Ucayali Facultad de Medicina Universidad Nacional de Ucayali Pucallpa Peru; 4 Universidad San Ignacio de Loyola, Lima, Peru. Universidad San Ignacio de Loyola Universidad San Ignacio de Loyola Lima Peru

**Keywords:** Radon, Lung Cancer, Environmental Health, Public Health, Health Surveillance

## Abstract

Radon is a globally recognized lung carcinogen and one of the main risk factors for lung cancer in non-smokers. In Peru, recent evidence indicates elevated radon concentrations in homes in Metropolitan Lima and a possible contribution to the increase in lung cancer, especially in women, although national evidence primarily comes from ecological studies and is still insufficient to establish causality. Regulatory, technical, and surveillance gaps persist that hinder its control. This special article presents a narrative review of international and national evidence and aims to examine the current situation of radon exposure in Peru, identify the regulatory and surveillance gaps that limit its control, and propose specific mitigation measures aligned with international recommendations.

## INTRODUCTION

Radon (²²²Rn) is a natural radioactive gas recognized as the second global cause of lung cancer and the first in non-smoking individuals ^1^. It originates from the disintegration of uranium present in rocks and soils and can accumulate in indoor environments, where its decay products emit alpha particles capable of damaging the respiratory epithelium ^2^^-^^4^.

Internationally, exposure has been documented in both occupational and residential settings, with variability determined by local geology and construction characteristics ^5^^,^^6^. In Latin America, including Peru, the absence of national radon maps and limited health surveillance hinder early detection and prevention ^7^.

In Metropolitan Lima, residential radon concentrations have been reported in multiple districts, with variability associated with the geological formations of the substrate ^8^^-^^10^. Recent research suggests a possible relationship between residential exposure and the increase in lung cancer in women, in a context where smoking has a low contribution to mortality ^11^^-^^13^; however, this relationship is supported so far by ecological and aggregated data, which is why a gap in national analytical evidence persists that this article seeks to contextualize.

The objective of this special article is to examine the current situation of radon exposure in Peru, identify the regulatory and surveillance gaps that limit its control, and propose specific mitigation measures aligned with international recommendations.

## METHODOLOGICAL APPROACH

A review of the scientific literature published between January 2010 and January 2026 was conducted in the PubMed, Scopus, and SciELO databases, in Spanish and English, supplemented with technical documents from the World Health Organization (WHO), the International Agency for Research on Cancer (IARC), the United States Environmental Protection Agency (EPA), and Peruvian regulations related to environmental health and cancer. Epidemiological studies, systematic reviews, meta-analyses, geological studies, and regulatory documents related to residential radon exposure and lung cancer were included; documents without full-text access or without a direct relationship to the study's objective were excluded. Additionally, a regulatory mapping was performed to identify regulatory gaps and opportunities for improvement in the Peruvian context. 124 records were identified; after removing duplicates and reviewing titles and abstracts, 58 documents were selected for the final analysis. Based on the evidence and the regulatory mapping, improvement proposals were developed, and the main challenges for their implementation were identified.

## RESULTS

### International evidence

International evidence consistently documents a sustained increase in lung cancer in nonsmoking individuals, with a higher incidence in women, particularly in East Asian populations ^14^^-^^17^. This epidemiological pattern suggests that, in addition to environmental factors such as residential radon exposure and other indoor pollutants, biological and genetic differences exist that increase female susceptibility.

In non-smoking women, lung cancer is more frequently associated with specific EGFR gene mutations, which reinforces its consideration as a differentiated clinical entity and underscores the need for preventive, diagnostic, and therapeutic strategies with a gender-specific approach ^18^. A possible association between radon exposure and a higher frequency of certain molecular alterations (e.g., EGFR mutations and ALK rearrangements) observed in patients with lung cancer not related to smoking has been postulated; however, the available findings are not statistically conclusive and current evidence does not allow establishing a direct causal relationship ^19^.

IARC classified radon as a group 1 carcinogen since 1988 ^20^. A meta-analysis estimated a 15% excess relative risk per 100 Bq/m³ ^21^. WHO explicitly promotes systematic radon measurement, the definition of a national reference level, and the inclusion of control measures in building codes, which underscores the need to integrate this scientific knowledge into public policy frameworks ^22^.

In the South American context, a systematic review that gathered 31 measurement studies in Central and South America concluded that Brazil, Argentina, and Peru concentrate most of the research, but that no country in the region yet has a national map of residential radon exposure or a unified legislative framework ^7^. In Brazil, an ecological time-series study in Rio Grande do Sul (2000-2021) observed that municipalities with a higher probability of radon exposure showed notable increases in lung cancer incidence and hospitalization, without significant differences in average rates, which illustrates both the exploratory value and the limitations of ecological designs in the region ^23^. These experiences —with geological, economic, and institutional conditions comparable to those in Peru— show that Peru shares the regional lag in systematic surveillance and constitute useful references for the design of a national program.

### Peruvian Evidence

In Peru, elevated concentrations have been recorded in various districts of Lima, with values that sometimes exceed 300 Bq/m³, particularly in areas influenced by granitic or alluvial deposits with high uranium content ^8^^,^^9^. The available Peruvian evidence continues to be limited and comes mainly from localized environmental measurements and ecological studies; consequently, there are still no national analytical studies that allow establishing causal associations between individual radon exposure and lung cancer.

The growing pattern of lung cancer in women in Metropolitan Lima ^11^^-^^13^^)^ and the projections of increased mortality towards 2030 ^24^^)^ suggest that environmental factors other than tobacco, among them radon, could be contributing to the risk ^25^, an interpretation that must be assumed with caution due to the risk of ecological fallacy.

To synthesize the process by which radon goes from the geological environment to become a lung carcinogen, a conceptual model is presented that integrates the described physical, biological, and public health mechanisms, from the emanation of radon in the soil to the development of lung cancer and surveillance and mitigation opportunities (figure 1).

**Figure 1:**
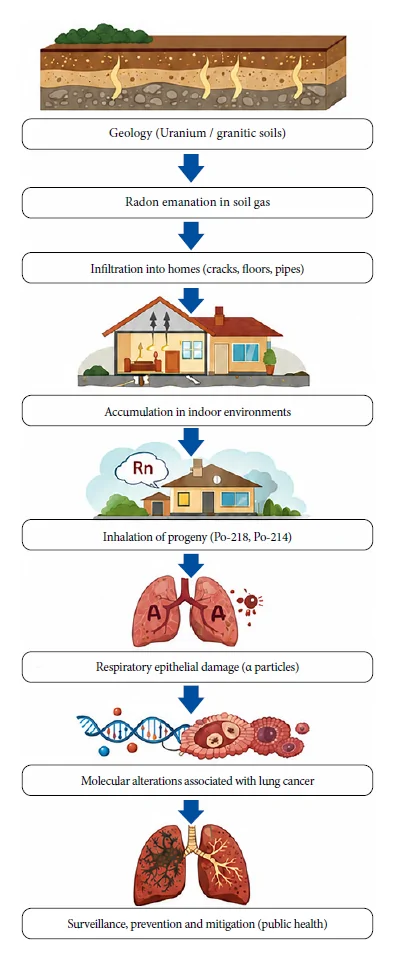
Radon exposure process and mechanisms related to lung cancer risk

The figure schematically represents the radon exposure pathway, from its entry and accumulation in indoor environments to the inhalation of its decay products and the possible damage to lung tissue. Po-218 (polonium-218) and Po-214 (polonium-214) are radioactive decay products of radon that emit alpha particles. Alpha particles (α) are ionizing radiations with high ionization capacity and short range, whose deposition in the respiratory epithelium can produce molecular alterations associated with lung cancer. Therefore, the surveillance, prevention, and mitigation of radon exposure are important measures for public health.

### Recommendations and challenges

International experience shows that the effective reduction of radon-associated risk is possible when scientific research, risk assessment, and intersectoral decision-making are integrated within strategic public health frameworks. The Peruvian case represents an opportunity to advance from evidence generation towards structured preventive policies against a preventable environmental risk.

The gap between scientific evidence and public policy is marked: while high-income countries have national maps, reference levels, and mitigation programs, in Peru, measures are practically nonexistent. Understanding exposure from an equity perspective allows us to recognize that risk is not uniformly distributed: the highest concentrations tend to coincide with homes with poorer ventilation or deficient infrastructure, disproportionately affecting vulnerable households. Integrating radon into national health priorities, with a gender and equity focus, is an urgent opportunity to reduce a preventable burden of cancer.

The WHO recommends a reference level of 100 Bq/m³ to minimize risk; however, it recognizes that, when this value cannot be achieved due to the country's specific circumstances, the selected level should not exceed 300 Bq/m³ ^22^. The International Commission on Radiological Protection reinforces this gradual approach by recommending to set the national reference level "as low as reasonably achievable" within the 100-300 Bq/m³ range, taking into account the prevailing economic and social circumstances ^20^. This gradual approach is relevant for Peru, where the obsolescence of D. S. N° 009-97-EM and technical and laboratory capacity limitations could make it difficult to reach 100 Bq/m³ in the short term. The definition of the reference level, as well as the regulatory update, should be based on an Ex Ante Regulatory Impact Analysis (RIA), an instrument that systematically evaluates the costs, benefits, and alternatives of a regulatory intervention, in accordance with D. S. N° 063-2021-PCM ^26^. This would allow for a phased adoption of a feasible value —for example, an initial target of 300 Bq/m³ with a progressive transition towards 100 Bq/m³— accompanied by measurement capabilities, institutional responsibilities, and progress indicators.

The proposed measures to strengthen radon control in Peru are summarized in table 1 and include the implementation of a national radon monitoring network, the update of the national reference level in accordance with WHO recommendations through an Ex Ante Regulatory Impact Analysis (RIA), the incorporation of preventive measures in the National Building Regulations, the strengthening of risk communication strategies aimed at the population, and the promotion of analytical research on residential radon exposure and lung cancer. Among the main challenges to implement these recommendations are the absence of accredited laboratories and technical capacity for radon measurement, the limited intersectoral coordination between the Ministry of Health, the Peruvian Institute of Nuclear Energy, the Ministry of Housing, Construction and Sanitation, the Presidency of the Council of Ministers, and the National Council for Science, Technology and Innovation (CONCYTEC), as well as insufficient sustained funding and the low level of public knowledge about this environmental risk. Recognizing these challenges is essential for the proposed actions to be feasible, gradual, and sustainable in the national context.

**Table 1:** Dimensions, WHO recommendations, situation in Peru, and proposed actions for radon control.

Dimension/ area	Recommendation of WHO	Current Situation (Peru)	Action/ Proposed recommendation	Responsible institution
Surveillance	National monitoring programs (WHO handbook on indoor radon)	There is no national surveillance program	Create a national radon monitoring network	MINSA-IPEN
Regulation	Optimal reference level of 100 Bq/m³ (up to 300 Bq/m³ if not achievable)	D. S. N° 009-97-EM: range of 200 to 600 Bq/m³	Align the reference level with WHO through RIA	MINSA-PCM
Construction	Preventive Measures in Buildings	Not incorporated into the National Building Regulations (RNE)	Update the RNE with prevention measures	MVCS
Public information	Awareness campaigns	Very limited	Educational strategy and risk communication	MINSA
Research	Population studies	Few studies	Financing national analytical research	CONCYTEC

Source: prepared by the authors.

WHO: World Health Organization, RIA: Ex Ante Regulatory Impact Analysis, MINSA: Ministry of Health, IPEN: Peruvian Institute of Nuclear Energy, MVCS: Ministry of Housing, Construction and Sanitation, PCM: Presidency of the Council of Ministers, CONCYTEC: National Council of Science, Technology and Innovation.

This article presents several limitations. First, the available Peruvian evidence is scarce and comes mainly from ecological studies and localized environmental measurements. Second, there are no national studies with individual assessment of exposure, residence time, smoking, occupation, or other confounding factors. Third, the observed associations between environmental radon levels and lung cancer must be interpreted with caution due to the risk of ecological fallacy. Finally, the absence of national surveillance programs limits the availability of representative information for the entire national territory.

## CONCLUSIONS

Radon constitutes an emerging and potentially preventable environmental risk in Peru. Although international evidence supports its association with lung cancer, national evidence continues to be insufficient to establish direct causal relationships and is based on ecological designs. The absence of systematic surveillance, updated national reference levels, and specific regulation constitutes a relevant gap for public health. Strengthening analytical research, implementing monitoring programs, and incorporating preventive measures into national regulations —supported by a regulatory impact analysis and by gradual and feasible goals— represent priority actions to reduce the risk associated with this environmental contaminant.

## References

[1] U.S. Environmental Protection Agency (2025). A citizen's guide to radon: the guide to protecting yourself and your family from radon. EPA 402/K-12/002.

[2] Rathebe PC, Mphaga KV, Masekameni DM (2025). Climate change and environmental radioactivity a review of studies on climate conditions in variation on indoor radon concentrations. Environ Monit Assess.

[3] Zhou J, Huang Q, Li W, Diao J, Lai L, Chen L (2026). Association of radon exposure with lung cancer serum biomarkers and computed tomography evidence from male miners. Environ Pollut.

[4] Kayouh N, Rabi R, Oufni L (2026). Modeling radon progeny deposition and dose in human airways using CFD Effects of respiratory patterns and environmental variables. Appl Radiat Isot.

[5] Torres-Cadavid E, Candal-Pedreira C, Ruano-Ravina A, Kelsey K, Martín-Gisbert L (2026). Is the Effect of Occupation on Lung Cancer Modified by Tobacco Use and Residential Radon Exposure Results of a Pooling Study. Arch Bronconeumol.

[6] Tejado-Ramos JJ, Alvarez-Toral A, Guillén J (2026). Evaluating overestimated radon risk in predictive maps A case study of cáceres municipality. J Environ Radioact.

[7] Giraldo-Osorio A, Ruano-Ravina A, Varela-Lema L, Barros-Dios JM, Pérez-Ríos M (2020). Residential radon in Central and South America a systematic review. Int J Environ Res Public Health.

[8] Pereyra P, López ME, Vilcapoma L (2015). Concentration measurements of radon 222 indoors in Lima-Peru. Int J Phys.

[9] Vilcapoma LL, López-Herrera ME, Pereyra P, Palacios DF, Pérez B, Rojas J (2019). Measurement of radon in soils of Lima City - Peru during the period 2016-2017. Earth Sci Res J.

[10] Khan MA, Khattak NU, Hanif M, Qadir A (2022). Assessment of soil-gas radon concentration over lithologies a case study from district Karak, Khyber Pakhtunkhwa, Pakistan. Environ Monit Assess.

[11] Ordoñez-Aquino C, Gonzales GF El radón, un gas radioactivo asociado a los gases de efecto invernadero, se encuentra en mayores concentraciones en distritos de Lima Metropolitana con alta incidencia de cáncer de pulmón.

[12] Ordoñez-Aquino C, Neciosup Delgado SP, Gonzales GF El radón, un gas radioactivo que se encuentra en mayores concentraciones en distritos de Lima Metropolitana con alta incidencia de cáncer de pulmón.

[13] Ordoñez-Aquino C, Gonzales GF, Vásquez-Velásquez C, Pereyra P, Cotrina E, Paredes S (2025). Dimorfismo sexual en la exposición a material particulado (PM2 5) y radón (²²²Rn) residencial, y su asociación con las tasas de incidencia de TBC y cáncer de pulmón en los 43 distritos de Lima Metropolitana. Rev Cuerpo Med HNAAA.

[14] Reichard JF, Barned S, Mendy A, Pinney SM (2025). Radon Exposure to the General Population of the Fernald Community Cohort. Atmosphere (Basel).

[15] Hong Y, Jang KS, Xie H (2025). Meta-analysis of the association between indoor environmental pollution and lung cancer risk in never-smokers. Am J Transl Res.

[16] Urrutia-Pereira M, Chatkin JM, Chong-Neto HJ, Solé D (2023). Radon exposure a major cause of lung cancer in nonsmokers. J Bras Pneumol.

[17] Martin-Gisbert L, Ruano-Ravina A, Varela-Lema L, Penabad M, Giraldo-Osorio A, Candal-Pedreira C (2023). Lung cancer mortality attributable to residential radon a systematic scoping review. J Expo Sci Environ Epidemiol.

[18] Zhao J, Li M, Niu L, Zhao Y, Song X (2025). Lung cancer in non-smoking women (LCINSW) from risk factors to precision therapy. Cancer Metastasis Rev.

[19] Ruano-Ravina A, Torres-Durán M, Kelsey KT, Parente-Lamelas I, Lei-ro-Fernández V, Abdulkader I (2016). Residential radon, EGFR mutations and ALK alterations in never-smoking lung cancer cases. Eur Respir J.

[20] International Commission on Radiological Protection (2014). Radiological protection against radon exposure ICRP Publication 126. Ann ICRP.

[21] Cheng ES, Egger S, Hughes S, Weber M, Steinberg J, Rahman B (2021). Systematic review and meta-analysis of residential radon and lung cancer in never-smokers. Eur Respir Rev.

[22] Organización Mundial de la Salud (2021). El radón y sus efectos en la salud.

[23] Jung JC, Costa JSD, Oliveira CHE, Filho OAS, Paniz VMV (2025). Temporal trends in lung cancer and radon gas exposure an ecological study in Rio Grande do Sul. Ecancermedicalscience.

[24] Solis-Sánchez G, Curisinche-Rojas M, Aliaga-Llerena KM, Muro-Cieza JF, Vidal-Anzardo MH, Instituto Nacional de Salud (2025). Tendencia de la tasa de mortalidad en el Perú por tipo de cáncer en el periodo 1999-2020 y predicción al año 2030: análisis de una fuente secundaria.

[25] Gaskin J, Coyle D, Whyte J, Krewksi D (2018). Global Estimate of Lung Cancer Mortality Attributable to Residential Radon. Environ Health Perspect.

[26] Presidencia del Consejo de Ministros (2021). Decreto Supremo N.° 063-2021-PCM, que aprueba el Reglamento que desarrolla el Marco Institucional que rige el Proceso de Mejora de la Calidad Regulatoria y establece los Lineamientos Generales para la aplicación del Análisis de Impacto Regulatorio Ex Ante.

