# Supplementary material for: Residential radon in Peru: challenges for health surveillance and lung cancer prevention
Source: Rev Peru Med Exp Salud Publica. 2026 Sep 14;43(3):397–401. doi: 10.17843/rpmesp.2026.433.16186 (PMC13718562; doi:10.17843/rpmesp.2026.433.16186)
Supplement: Translation

# Translation: Radón residencial en el Perú: desafíos para la vigilancia sanitaria y la prevención del cáncer de pulmón

**Authors:** Carol Ordoñez-Aquino, Diego Venegas Ojeda, Jhony A. De La Cruz-Vargas, Gustavo F. Gonzales

**Affiliations:** 1 Programa de Doctorado en Ciencias de la Salud, Facultad de Medicina, Universidad Nacional Mayor de San Marcos, Lima, Perú.; 2 Facultad de Ciencias e Ingeniería, Universidad Peruana Cayetano Heredia, Lima, Perú.; 3 Facultad de Medicina, Universidad Nacional de Ucayali, Pucallpa, Perú.; 4 Universidad San Ignacio de Loyola, Lima, Perú.

**Keywords:** Radón, Cáncer de Pulmón, Salud Ambiental, Salud Pública, Vigilancia Sanitaria

## Abstract

El radón es un carcinógeno pulmonar reconocido a nivel mundial y uno de los principales factores de riesgo de cáncer de pulmón en personas no fumadoras. En el Perú, la evidencia reciente indica concentraciones elevadas de radón en viviendas de Lima Metropolitana y una posible contribución al incremento del cáncer de pulmón, especialmente en mujeres**,** aunque la evidencia nacional proviene principalmente de estudios ecológicos y aún es insuficiente para establecer causalidad. Persisten brechas normativas, técnicas y de vigilancia que dificultan su control. Este artículo especial presenta una revisión narrativa de la evidencia internacional y nacional y tiene como objetivo examinar la situación actual de la exposición al radón en el Perú, identificar las brechas regulatorias y de vigilancia que limitan su control y proponer medidas específicas de mitigación alineadas con recomendaciones internacionales.

## INTRODUCCIÓN

El radón (²²²Rn) es un gas radiactivo natural reconocido como la segunda causa global de cáncer de pulmón y la primera en personas no fumadoras ^1^. Se origina por la desintegración del uranio presente en rocas y suelos y puede acumularse en ambientes interiores, donde sus productos de decaimiento emiten partículas alfa capaces de dañar el epitelio respiratorio ^2^^-^^4^.

A nivel internacional, la exposición se ha documentado tanto en contextos ocupacionales como residenciales, con variabilidad determinada por la geología local y las características constructivas ^5^^,^^6^. En América Latina, incluido el Perú, la ausencia de mapas nacionales de radón y la limitada vigilancia sanitaria dificultan la detección temprana y la prevención ^7^.

En Lima Metropolitana se han reportado concentraciones residenciales de radón en múltiples distritos, con variabilidad asociada a las formaciones geológicas del sustrato ^8^^-^^10^. Investigaciones recientes sugieren una posible relación entre la exposición residencial y el incremento del cáncer de pulmón en mujeres, en un contexto donde el tabaquismo tiene baja contribución a la mortalidad ^11^^-^^13^; no obstante, dicha relación se sustenta hasta ahora en datos ecológicos y agregados, por lo que persiste una brecha de evidencia analítica nacional que este artículo busca contextualizar.

El objetivo de este artículo especial es examinar la situación actual de la exposición al radón en el Perú, identificar las brechas regulatorias y de vigilancia que limitan su control y proponer medidas específicas de mitigación alineadas con recomendaciones internacionales.

## ABORDAJE METODOLÓGICO

Se realizó una revisión de la literatura científica publicada entre enero de 2010 y enero de 2026 en las bases de datos PubMed, Scopus y SciELO, en español e inglés, complementada con documentos técnicos de la Organización Mundial de la Salud (OMS), la Agencia Internacional para la Investigación del Cáncer (IARC), la Agencia de Protección Ambiental de los Estados Unidos (EPA) y normativa peruana relacionada con salud ambiental y cáncer. Se incluyeron estudios epidemiológicos, revisiones sistemáticas, metaanálisis, estudios geológicos y documentos normativos relacionados con exposición residencial al radón y cáncer de pulmón; se excluyeron documentos sin acceso al texto completo o sin relación directa con el objetivo del estudio. Adicionalmente, se realizó un mapeo normativo para identificar brechas regulatorias y oportunidades de mejora en el contexto peruano. Se identificaron 124 registros; tras eliminar duplicados y revisar títulos y resúmenes, se seleccionaron 58 documentos para el análisis final. A partir de la evidencia y del mapeo normativo se elaboraron las propuestas de mejora y se identificaron los principales desafíos para su implementación.

## RESULTADOS

### Evidencia internacional

La evidencia internacional documenta de forma consistente un aumento sostenido del cáncer de pulmón en personas no fumadoras, con una mayor incidencia en mujeres, particularmente en poblaciones de Asia Oriental ^14^^-^^17^. Este patrón epidemiológico sugiere que, además de factores ambientales como la exposición residencial al radón y a otros contaminantes intradomiciliarios, existen diferencias biológicas y genéticas que incrementan la susceptibilidad femenina.

En mujeres no fumadoras, el cáncer de pulmón se asocia con mayor frecuencia a mutaciones específicas del gen EGFR, lo que refuerza su consideración como una entidad clínica diferenciada y subraya la necesidad de estrategias preventivas, diagnósticas y terapéuticas con enfoque de género ^18^. Se ha postulado una posible asociación entre la exposición al radón y una mayor frecuencia de determinadas alteraciones moleculares (p. ej., mutaciones en EGFR y reordenamientos de ALK) observadas en pacientes con cáncer de pulmón no relacionado al tabaquismo; sin embargo, los hallazgos disponibles no son estadísticamente concluyentes y la evidencia actual no permite establecer una relación causal directa ^19^.

La IARC clasificó el radón como carcinógeno del grupo 1 desde 1988 ^20^. Un metaanálisis estimó un exceso de riesgo relativo de 15% por cada 100 Bq/m³ ^21^. La OMS promueve explícitamente la medición sistemática del radón, la definición de un nivel de referencia nacional y la inclusión de medidas de control en las normas constructivas, lo que subraya la necesidad de integrar este conocimiento científico en marcos de política pública ^22^.

En el contexto sudamericano, una revisión sistemática que reunió 31 estudios de medición en Centro y Sudamérica concluyó que Brasil, Argentina y el Perú concentran la mayor parte de la investigación, pero que ningún país de la región dispone aún de un mapa nacional de exposición residencial al radón ni de un marco legislativo unificado ^7^. En Brasil, un estudio ecológico de series temporales en Rio Grande do Sul (2000-2021) observó que los municipios con mayor probabilidad de exposición a radón presentaron incrementos notables en la incidencia y la hospitalización por cáncer de pulmón, sin diferencias significativas en las tasas promedio, lo que ilustra tanto el valor exploratorio como las limitaciones de los diseños ecológicos en la región ^23^. Estas experiencias -con condiciones geológicas, económicas e institucionales comparables a las peruanas- evidencian que el Perú comparte el rezago regional en vigilancia sistemática y constituyen referentes útiles para el diseño de un programa nacional.

### Evidencia peruana

En el Perú, se han registrado concentraciones elevadas en diversos distritos de Lima, con valores que superan por momentos los 300 Bq/m³, particularmente en zonas influenciadas por depósitos graníticos o aluviales de alto contenido de uranio ^8^^,^^9^. La evidencia peruana disponible continúa siendo limitada y proviene principalmente de mediciones ambientales localizadas y estudios ecológicos; en consecuencia, no existen aún estudios analíticos nacionales que permitan establecer asociaciones causales entre la exposición individual al radón y el cáncer de pulmón.

El patrón creciente de cáncer de pulmón en mujeres en Lima Metropolitana ^11^^-^^13^^)^ y las proyecciones de incremento de mortalidad hacia 2030 ^24^^)^ sugieren que factores ambientales distintos al tabaco, entre ellos el radón, podrían estar contribuyendo al riesgo ^25^, interpretación que debe asumirse con cautela por el riesgo de falacia ecológica.

Para sintetizar el proceso mediante el cual el radón pasa del entorno geológico a convertirse en un carcinógeno pulmonar, se presenta un modelo conceptual que integra los mecanismos físicos, biológicos y de salud pública descritos, desde la emanación del radón en el suelo hasta el desarrollo del cáncer de pulmón y las oportunidades de vigilancia y mitigación (figura 1).

**Figura 1:** Proceso de exposición al radón y mecanismos relacionados con el riesgo de cáncer de pulmón.

La figura representa esquemáticamente la ruta de exposición al radón, desde su ingreso y acumulación en ambientes interiores hasta la inhalación de sus productos de desintegración y el posible daño del tejido pulmonar. El Po-218 (polonio-218) y el Po-214 (polonio-214) son productos radiactivos de desintegración del radón que emiten partículas alfa. Las partículas alfa (α) son radiaciones ionizantes de alta capacidad de ionización y corto alcance, cuya deposición en el epitelio respiratorio puede producir alteraciones moleculares asociadas al cáncer de pulmón. Por ello, la vigilancia, prevención y mitigación de la exposición al radón son medidas de importancia para la salud pública.

### Recomendaciones y desafíos

La experiencia internacional muestra que la reducción efectiva del riesgo asociado al radón es posible cuando se integran la investigación científica, la evaluación de riesgo y la toma de decisiones intersectoriales dentro de marcos estratégicos de salud pública. El caso peruano representa una oportunidad para avanzar desde la generación de evidencia hacia políticas preventivas estructuradas frente a un riesgo ambiental prevenible.

La brecha entre la evidencia científica y la política pública es marcada: mientras los países de altos ingresos cuentan con mapas nacionales, niveles de referencia y programas de mitigación, en el Perú las medidas son prácticamente inexistentes. Comprender la exposición desde una perspectiva de equidad permite reconocer que el riesgo no se distribuye de manera uniforme: las concentraciones más altas tienden a coincidir con viviendas de menor ventilación o infraestructura deficiente, afectando de forma desproporcionada a hogares vulnerables**.** Integrar el radón en las prioridades sanitarias nacionales, con enfoque de género y de equidad, es una oportunidad urgente para reducir una carga prevenible de cáncer.

La OMS recomienda un nivel de referencia de 100 Bq/m³ para minimizar el riesgo; sin embargo, reconoce que, cuando este valor no pueda alcanzarse por las circunstancias específicas del país, el nivel seleccionado no debe superar los 300 Bq/m³ ^22^**.** La Comisión Internacional de Protección Radiológica refuerza este enfoque gradual al recomendar fijar el nivel de referencia nacional «tan bajo como sea razonablemente alcanzable» dentro del rango de 100-300 Bq/m³, tomando en cuenta las circunstancias económicas y sociales prevalecientes ^20^. Esta gradualidad es pertinente para el Perú, donde el desfase del D. S. N° 009-97-EM y las limitaciones técnicas y de capacidad de laboratorio podrían dificultar alcanzar los 100 Bq/m³ en el corto plazo. La definición del nivel de referencia, así como la actualización normativa, debería sustentarse en un Análisis de Impacto Regulatorio Ex Ante (AIR), instrumento que evalúa de manera sistemática los costos, beneficios y alternativas de una intervención regulatoria, conforme al D. S. N° 063-2021-PCM ^26^. Ello permitiría adoptar de forma escalonada un valor factible -por ejemplo, una meta inicial de 300 Bq/m³ con transición progresiva hacia 100 Bq/m³- acompañado de capacidades de medición, responsables institucionales e indicadores de progreso.

Las medidas propuestas para fortalecer el control del radón en el Perú se resumen en la tabla 1 e incluyen la implementación de una red nacional de monitoreo de radón, la actualización del nivel nacional de referencia conforme a las recomendaciones de la OMS mediante un Análisis de Impacto Regulatorio Ex Ante (AIR), la incorporación de medidas preventivas en el Reglamento Nacional de Edificaciones, el fortalecimiento de las estrategias de comunicación del riesgo dirigidas a la población y el impulso de investigaciones analíticas sobre exposición residencial de radón y el cáncer de pulmón. Entre los principales desafíos para implementar estas recomendaciones destacan la ausencia de laboratorios acreditados y de capacidad técnica para la medición de radón, la limitada coordinación intersectorial entre el Ministerio de Salud, el Instituto Peruano de Energía Nuclear, el Ministerio de Vivienda, Construcción y Saneamiento, la Presidencia del Consejo de Ministros y el Consejo Nacional de Ciencia, Tecnología e Innovación (CONCYTEC), así como la insuficiencia de financiamiento sostenido y el bajo nivel de conocimiento público sobre este riesgo ambiental. Reconocer estos desafíos resulta indispensable para que las acciones propuestas sean factibles, graduales y sostenibles en el contexto nacional.

**Tabla 1:** Dimensiones, recomendaciones de la OMS, situación en el Perú y acciones propuestas para el control del radón.

Dimensión/ área	Recomendación de OMS	Situación actual (Perú)	Acción/ recomendación propuesta	Institución responsable
Vigilancia	Programas nacionales de monitoreo (Manual de la OMS sobre el radón en interiores)	No existe un programa nacional de vigilancia	Crear una red nacional de monitoreo de radón	MINSA-IPEN
Regulación	Nivel de referencia óptimo de 100 Bq/m³ (hasta 300 Bq/m³ si no es alcanzable)	D. S. N° 009-97-EM: rango de 200 a 600 Bq/m³	Alinear el nivel de referencia con la OMS mediante AIR	MINSA-PCM
Construcción	Medidas preventivas en edificaciones	No incorporado al Reglamento Nacional de Edificaciones (RNE)	Actualizar el RNE con medidas de prevención	MVCS
Información pública	Campañas de sensibilización	Muy limitadas	Estrategia educativa y comunicación de riesgo	MINSA
Investigación	Estudios poblacionales	Escasos estudios	Financiar investigación analítica nacional	CONCYTEC

Fuente: elaboración propia de los autores.

OMS: Organización Mundial de la Salud, AIR: Análisis de Impacto Regulatorio Ex Ante, MINSA: Ministerio de Salud, IPEN: Instituto Peruano de Energía Nuclear, MVCS: Ministerio de Vivienda, Construcción y Saneamiento, PCM: Presidencia del Consejo de Ministros, CONCYTEC: Consejo Nacional de Ciencia, Tecnología e Innovación.

Este artículo presenta varias limitaciones. Primero, la evidencia peruana disponible es escasa y proviene principalmente de estudios ecológicos y mediciones ambientales localizadas. Segundo, no existen estudios nacionales con evaluación individual de la exposición, el tiempo de residencia, el tabaquismo, la ocupación u otros factores de confusión. Tercero, las asociaciones observadas entre los niveles ambientales de radón y el cáncer de pulmón deben interpretarse con cautela debido al riesgo de falacia ecológica. Finalmente, la ausencia de programas nacionales de vigilancia limita la disponibilidad de información representativa para todo el territorio nacional.

## CONCLUSIONES

El radón constituye un riesgo ambiental emergente y potencialmente prevenible en el Perú. Aunque la evidencia internacional respalda su asociación con el cáncer de pulmón, la evidencia nacional continúa siendo insuficiente para establecer relaciones causales directas y se basa en diseños ecológicos. La ausencia de vigilancia sistemática, de niveles de referencia nacionales actualizados y de regulación específica constituye una brecha relevante para la salud pública. Fortalecer la investigación analítica, implementar programas de monitoreo e incorporar medidas preventivas en la normativa nacional -sustentadas en un análisis de impacto regulatorio y en metas graduales y factibles**-** representan acciones prioritarias para reducir el riesgo asociado a este contaminante ambiental.
